# The EANM practice guidelines for parathyroid imaging

**DOI:** 10.1007/s00259-021-05334-y

**Published:** 2021-04-10

**Authors:** Petra Petranović Ovčariček, Luca Giovanella, Ignasi Carrió Gasset, Elif Hindié, Martin W. Huellner, Markus Luster, Arnoldo Piccardo, Theresia Weber, Jean-Noël Talbot, Frederik Anton Verburg

**Affiliations:** 1 EANM Thyroid Committee, Vienna, Austria; 2 Department of Oncology and Nuclear medicine, University Hospital Center “Sestre milosrdnice”, Zagreb, Croatia; 3 Clinic for Nuclear Medicine and Competence Centre for Thyroid Diseases, Imaging Institute of Southern Switzerland, Bellinzona, Switzerland; 4 Clinic for Nuclear Medicine and Interdisciplinary Thyroid Centre, University Hospital and University of Zurich, Zurich, Switzerland; 5 Department of Nuclear Medicine, Hospital Sant Pau and Autonomous University of Barcelona, Barcelona, Spain; 6 Department of Nuclear Medicine, Bordeaux Hospital and University, Bordeaux, France; 7 Department of Nuclear Medicine, University Hospital and University of Zurich, Zurich, Switzerland; 8 Department of Nuclear Medicine, University Hospital Marburg, Marburg, Germany; 9 Department of Nuclear Medicine, E.O. Ospedali Galliera, Genoa, Italy; 10 Department of Endocrine Surgery, Katholisches Klinikum Mainz, Mainz, Germany; 11 Nuclear Medicine, Hospital Tenon APHP and Sorbonne University, Paris, France; 12 Department of Radiology and Nuclear Medicine, Erasmus MC, Rotterdam, The Netherlands

**Keywords:** Parathyroid scintigraphy, Hyperparathyroidism, [^99m^Tc]Tc-MIBI, [^99m^Tc]Tc-tetrofosmin, SPECT/CT, Dual-phase scintigraphy, Dual-tracer scintigraphy, ^18^F-labeled choline analogues, [^11^C]CH, [^11^C]MET, PET/CT, Cervical ultrasonography, 4D-CT, MRI

## Abstract

**Introduction:**

Nuclear medicine parathyroid imaging is important in the identification of hyperfunctioning parathyroid glands in primary hyperparathyroidism (pHPT), but it may be also valuable before surgical treatment in secondary hyperparathyroidism (sHPT). Parathyroid radionuclide imaging with scintigraphy or positron emission tomography (PET) is a highly sensitive procedure for the assessment of the presence and number of hyperfunctioning parathyroid glands, located either at typical sites or ectopically. The treatment of pHPT is mostly directed toward minimally invasive parathyroidectomy, especially in cases with a single adenoma. In experienced hands, successful surgery depends mainly on the exact preoperative localization of one or more hyperfunctioning parathyroid adenomas. Failure to preoperatively identify the hyperfunctioning parathyroid gland challenges minimally invasive parathyroidectomy and might require bilateral open neck exploration.

**Methods:**

Over a decade has now passed since the European Association of Nuclear Medicine (EANM) issued the first edition of the guideline on parathyroid imaging, and a number of new insights and techniques have been developed since. The aim of the present document is to provide state-of-the-art guidelines for nuclear medicine physicians performing parathyroid scintigraphy, single-photon emission computed tomography/computed tomography (SPECT/CT), positron emission tomography/computed tomography (PET/CT), and positron emission tomography/magnetic resonance imaging (PET/MRI) in patients with pHPT, as well as in those with sHPT.

**Conclusion:**

These guidelines are written and authorized by the EANM to promote optimal parathyroid imaging. They will assist nuclear medicine physicians in the detection and correct localization of hyperfunctioning parathyroid lesions.

## Preamble

The European Association of Nuclear Medicine (EANM) is a professional non-profit medical association that facilitates communication worldwide among individuals pursuing clinical and research excellence in nuclear medicine. The EANM was founded in 1985.

These guidelines are intended to assist practitioners in providing appropriate nuclear medicine care for patients. They are not inflexible rules or requirements of practice and are not intended, nor should they be used, to establish a legal standard of care.

The ultimate judgment regarding the propriety of any specific procedure or course of action must be made by medical professionals taking into account the unique circumstances of each case. Thus, there is no implication that an approach differing from the guidelines, standing alone, is below the standard of care. To the contrary, a conscientious practitioner may responsibly adopt a course of action different from that set out in the guidelines when, in the reasonable judgment of the practitioner, such course of action is indicated by the condition of the patient, limitations of available resources, or advances in knowledge or technology subsequent to publication of the guidelines. The practice of medicine involves not only the science but also the art of dealing with the prevention, diagnosis, alleviation, and treatment of disease.

The variety and complexity of human conditions make it impossible to always reach the most appropriate diagnosis or to predict with certainty a particular response to treatment. Therefore, it should be recognized that adherence to these guidelines will not ensure an accurate diagnosis or a successful outcome.

All that should be expected is that the practitioner will follow a reasonable course of action based on current knowledge, available resources, and the needs of the patient to deliver effective and safe medical care. The sole purpose of these guidelines is to assist practitioners in achieving this objective.

## Introduction

Primary hyperparathyroidism is an endocrine disorder caused by one or more hyperfunctioning parathyroid glands. The suspicion of pHPT is typically raised by the detection of hypercalcemia and/or its associated symptoms. Parathyroid hormone (PTH) serum concentration is elevated, or within normal limits but inappropriate considering the presence of hypercalcemia. A rare type of pHPT is normocalcemic hyperparathyroidism presenting with normal serum calcium levels but an elevated PTH concentration [1].

Primary hyperparathyroidism is the third most common endocrine disease [2], affecting women 2–3 times more often than men [3]. It is caused by the hyperfunction of one or more parathyroid glands, which are overproducing PTH. Hyperfunctioning parathyroid glands are solitary adenomas in the majority of cases. Approximately 15–20% of cases are caused by multiglandular disease (MGD) [4], i.e., multiple adenomas or hyperplasia, while parathyroid carcinoma accounts for less than 1% of cases. The majority of pHPT cases (95%) occur sporadically, but approximately 5% are part of hereditary syndromes, such as multiple endocrine neoplasia types 1, 2, and 4 (MEN-1, MEN-2, and MEN-4) and hyperparathyroidism-jaw tumor syndrome, or as part of non-syndromic familial pHPT [5].

Familial hypocalciuric hypercalcemia, a potential differential diagnosis to pHPT, has to be recognized as it usually does not require surgery.

Secondary hyperparathyroidism is a disorder characterized by increased serum PTH levels due to parathyroid gland hyperplasia, triggered by hypocalcemia, hyperphosphatemia, or decreased serum vitamin D concentration. The most common causes of sHPT are vitamin D deficiency and chronic renal disease. Sometimes pHPT and vitamin D deficiency occur simultaneously, which results in decreased or normal serum calcium levels. Rarer causes are long-term treatment with lithium or magnesium, intestinal malabsorption or malnutrition, or sunitinib treatment.

The therapy of sHPT depends on the primary cause. In the case of renal HPT, therapy mainly consists of a restriction of dietary intake together with medical treatment with calcimimetics, vitamin D analogues, and phosphorus binders [6]. Still, parathyroidectomy is required in patients with renal sHPT refractory to medical treatment, i.e., in approximately 15% of patients after 10 years and 38% of patients after 20 years of renal dialysis therapy [7].

Paraneoplastic hyperparathyroidism is rare. In most cases it is due to parathyroid hormone-related peptide (PTH-rp) production and the serum level of PTH is low in relation to hypercalcemia. In rare cases, the culprit cancer produces PTH or both PTH and PTH-rp, while no hyperfunctioning parathyroid gland is present [8]. Most commonly, the PTH-rp secreting malignancies include squamous cell carcinoma (lung, head and neck, cervix) and some other solid tumors.

Parathyroid glands are typically located along both thyroid lobes. The superior parathyroid glands develop from the 4th pharyngeal pouch and descend with the thyroid. They are located dorsal to the middle and upper poles of the thyroid, and above the inferior thyroid artery. The inferior parathyroid glands develop from the 3rd pouch and descend with the thymus to ultimately remain close to the lower thyroid pole, and more laterally [9]. Superior and inferior parathyroid glands have a different relationship to the recurrent laryngeal nerve, with the superior ones lying posterior to it, and the inferior ones anterior to it.

The incidence of ectopic parathyroid glands is approximately 16% [10]. An ectopically located gland may be one of the four standard parathyroid glands or a supernumerary gland. Most individuals (84%) have four parathyroid glands, two superior and two inferior glands, while supernumerary glands exist in 3 to 13% of individuals [11]. The total number of parathyroid glands in an individual with supernumerary glands may vary from 5 to 8 in number [12]. Rarely, in less than 3% of cases, individuals may have only 3 glands [11].

Acquired ectopy of upper parathyroid glands is frequently encountered along the tracheoesophageal groove or more rarely retroesophageally. They are usually in a lower position than expected (prolapsed glands) [13]. Moreover, ectopic glands can be located anywhere along the migratory path [14], from the carotid bifurcation to the pericardium. Ectopic sites, therefore, include but are not limited to, a high cervical position, the carotid sheath, an intrathyroidal location, intrathymic location, mediastinal location, or the paraesophageal region [15]. Very rarely, they can also be located in the pericardium [16, 17]. However, the most common location for ectopic upper parathyroid glands is along the esophagus, while ectopic lower parathyroid glands most often have an intrathymic location [9].

Histologically, parathyroid glands consist of a chief, oxyphil, transitional oxyphil, and water-clear cells. Chief cells are the most abundant cells [18]. They have a secretory function and are responsible for synthesizing and secreting PTH. Oxyphil cells are rich in mitochondria and their function is still unclear [19]. The function of water-clear cells is not clearly understood as well.

Owing to the abundance of mitochondria within the oxyphil cells, there is a high accumulation of [^99m^Tc]Tc-MIBI and [^99m^Tc]Tc-tetrofosmin in hyperfunctioning parathyroid glands.

Radionuclide parathyroid imaging reveals hyperfunctioning parathyroid glands located at typical sites as well as ectopically. Using planar scintigraphy or SPECT, sensitivity is greater for adenomas compared with hyperplasia. Hyperplastic parathyroid glands are usually smaller than adenomas, which is associated with decreased sensitivity of radionuclide imaging [20]. Other factors may also contribute to reduced sensitivity for MGD (e.g., tracer washout from some lesions in dual-phase imaging, the inhibitory effect of hypercalcemia, etc.). However, it is important to recognize MGD, as it holds implications for a successful surgery. Each subsequent surgery in a postoperative neck is technically challenging and carries an additional risk of complications, including failure to remove the culprit gland(s).

For the reasons stated above, it is necessary to identify the most sensitive and specific diagnostic procedure to detect hyperfunctioning parathyroid glands. The currently most widespread preoperative imaging procedure is the combination of [^99m^Tc]Tc-MIBI scintigraphy and cervical ultrasonography (cUS), yielding a sensitivity of 81–95% [21, 22], which is mainly contributed by [^99m^Tc]Tc-MIBI scintigraphy. Cervical ultrasonography can be combined with targeted fine-needle aspiration (FNA) cytology and detection of PTH in the aspirate (FNA-PTH) in order to increase specificity and confirm the diagnosis of hyperfunctioning parathyroid lesions in sonographically accessible locations [23]. Fine-needle aspiration cytology is recommended in highly selected cases, e.g., atypical cUS presentation and uninformative [^99m^Tc]Tc-MIBI. In case of negative standard imaging, second-line imaging may be performed, such as N-[(^18^F)fluoromethyl]-2-hydroxy-N,N-dimethylethanaminium ([^18^F]FCH or [^18^F]fluorocholine) PET/CT, L-[*methy*l-^11^C]methionine ([^11^C]MET) PET/CT, [^11^C]2-hydroxy-N,N,N-trimethylethanamium ([^11^C]CH) PET/CT, so-called four-dimensional computed tomography (4D-CT), MRI, [^18^F]fluorocholine PET/4D-CT or [^18^F]fluorocholine PET/MRI. [^18^F]fluorocholine PET/CT is also considered a potential “alternative” first-line method whenever possible, as it appears to be an effective technique even in patients with negative or equivocal standard imaging findings. It is particularly useful in patients with sHPT [24–26]. Before revision surgery and/or in the case of negative imaging techniques, invasive techniques, such as selective venous sampling or selective arteriography, may be performed.

## Goals

The EANM practice guidelines for parathyroid imaging are written for nuclear medicine practitioners to promote the use of optimal parathyroid imaging based on current knowledge. The purpose of these recommendations is to assist in the performance, the correct interpretation, and reporting of the results of the parathyroid imaging.

## Background

Primary hyperparathyroidism is defined as high serum PTH levels due to the presence of enlarged hyperfunctioning parathyroid gland(s). It is the most common cause of elevated calcium levels in the ambulatory setting [3], although calcemia in some cases may be normal. Rarely, PTH levels are normal with concomitant hypercalcemia. Primary hyperparathyroidism represents a considerable public health problem due to its high incidence and repercussions to patients’ health. Therapy primarily consists of the surgical removal of the hyperfunctioning parathyroid gland(s) [27]. Symptomatic patients require treatment and generally undergo parathyroidectomy, which is the only definitive therapy. Surgery is also an important option in asymptomatic patients who can develop hypercalcemia and target organ involvement later in life [28]. If surgery is indicated, preoperative localization of the hyperfunctioning gland(s) allows for selective parathyroidectomy, reducing operation time, complications and hospitalization, improving postoperative recovery, and resulting in a better cosmetic outcome, and greater patient satisfaction [29, 30]. Nuclear medicine techniques and cUS are crucial in identifying and localizing hyperfunctioning parathyroid glands. Both methods have advantages, as well as limitations. The advantage of radionuclide parathyroid imaging over cUS lies in the identification of ectopic glands [31], as well as in easier recognition of posteriorly located upper glands [32]. Arguably it also enables an easier recognition of typically located hyperfunctioning parathyroid glands in the background of thyroiditis. It is known that thyroiditis is usually followed by enlarged lymph nodes that are sometimes imitating hyperfunctioning parathyroid glands on cUS [33]. On the other hand, suspected hyperfunctioning parathyroid glands in the neck on radionuclide imaging can be further examined with cUS in order to avoid false-positive results. In patients with multinodular goiter, [^99m^Tc]Tc-MIBI scintigraphy might be false negative, and cUS is mandatory [34]. In these cases, dual-tracer methods and [^18^F]fluorocholine PET/CT are useful as well [35]. Additionally, cUS is a valuable method for excluding coexisting thyroid pathology in most cases, although in toxic multinodular goiter or thyroid remnants after hemithyroidectomy dual-tracer methods are helpful as well. If a hyperfunctioning parathyroid gland is identified during cUS, radionuclide imaging may still prove to be useful because of possible undetected MGD, as well as ectopic glands. For the reasons stated above, in addition to radionuclide parathyroid gland imaging, using SPECT and/or PET tracers, these guidelines will also discuss ultrasound of the parathyroid glands as the main complementary diagnostic imaging modality. Additionally, the use of 4D CT, MRI, and invasive techniques in identifying abnormal parathyroid glands will be addressed in brief.

## Indications

Radionuclide parathyroid imaging is used for the localization of one or more hyperfunctioning parathyroid glands in patients with pHPT, and not for the diagnosis of hyperparathyroidism (HPT). It might be also valuable before surgical treatment in sHPT due to possible detection of ectopic and supernumerary glands, as well as a parathyroid gland with the lowest [^99m^Tc]Tc-MIBI uptake which may be partially autografted or preserved [36, 37]. Périé et al. recommend a combination of cUS and dual-tracer scintigraphy for routine use to localize hyperfunctioning parathyroid glands in patients with renal HPT undergoing surgical treatment [38]. However, surgeons usually do not order [^99m^Tc]Tc-MIBI scintigraphy before primary surgery for renal hyperparathyroidism, and bilateral open neck exploration is considered in these patients. Still, radionuclide imaging may be helpful in cases of revision surgery. In such circumstances, a combination of imaging modalities is often required [37].

Radionuclide imaging is useful preoperatively to guide the surgeon toward the exact location of one or more hyperfunctioning parathyroid glands, especially in the case of ectopic glands that cannot be visualized with cUS. Correct preoperative localization may shorten the duration of surgery, which nowadays mostly consists of minimally invasive parathyroidectomy combined with intraoperative PTH determination, particularly in single-gland disease. In the past, bilateral open neck exploration was the standard surgical approach. However, both techniques are appropriate and accomplish high cure rates [39]. Excision of all hyperfunctioning parathyroid glands is pivotal for successful parathyroidectomy.

Radionuclide parathyroid imaging is especially useful in patients with recurrent disease because the second operation is technically more challenging than the first one, and hence a precise preoperative localization of hyperfunctioning parathyroid gland(s) is important.

Radionuclide parathyroid imaging is also useful in conjunction with cUS, foremost in patients with hereditary disorders, such as MEN-1, MEN-2, and MEN-4, although sensitivity for hyperplasia is lower compared with sporadic adenoma. In this context, PET/CT might be preferred, owing to its higher spatial resolution [40]. The identification of hyperfunctioning glands can be supported by a γ-probe during surgery, especially in patients who underwent previous surgical treatment [41].

## Precautions

It is necessary to exclude pregnancy in women of childbearing age.

In pregnant patients, radionuclide imaging should only be performed if deemed absolutely necessary, as decided by a multidisciplinary team. Otherwise, it should be postponed after pregnancy. Instead, cUS and MRI may be preferred in this case.

For more information, we advise to consult the American College of Radiology (ACR) Practice Guideline for imaging pregnant or potentially pregnant adolescents and women with ionizing radiation [42].

In breastfeeding women, it is recommended to consult the International Commission on Radiological Protection (ICRP) Publication 128: Radiation Dose to Patients from Radiopharmaceuticals: A Compendium of Current Information Related to Frequently Used Substances [43]. When using ^99m^Tc-labeled radiopharmaceuticals, interruption of breastfeeding is not essential if free pertechnetate is absent in the radiopharmaceutical. However, a pause of 24 h during which expressed feeds are discarded is recommended in some summaries of product characteristics (SmPC) of ^99m^Tc labeled radiopharmaceuticals with marketing authorization. In the case of Na[^99m^Tc]TcO_4_, which is used for subtraction imaging, a 12 h pause is recommended. For Na[^123^I]I, breastfeeding interruption for > 3 weeks is advised due to the risk of other iodine isotope contamination. However, if the product has high purity (> 99.65%), the pause may be shorter. In the SmPC of some Na[^123^I]I preparations, 1–3 days pause is recommended, and breastfeeding can be restarted when the activity level in the milk has reduced so that it will not result in a radiation dose to the child greater than 1 mSv. To be on the safe side we recommend a 3-day pause. It is not known whether [^11^C]CH, ^18^F-labeled choline analogues, and [^11^C]MET are excreted in breast milk. Still, for ^11^C-labeled radiopharmaceuticals, interruption is not essential due to their short physical half-life. No recommendation concerning the pause in lactation for ^18^F-labeled choline analogues is available in ICRP publication 128, but it may be found in the SmPC of the ^18^F-labeled choline analogues preparation with a marketing authorization. A 12 h pause is recommended in some SmPCs.

## Qualifications and responsibilities of personnel

Nuclear medicine physicians, technicians and all other staff involved in performing and reporting parathyroid radionuclide imaging should be qualified according to applicable laws and regulations, and individual responsibilities should be clearly documented.

## Useful clinical information for optimal imaging and interpretation

A request for parathyroid radionuclide imaging must be submitted to a nuclear medicine physician. It should provide sufficient data to allow the responsible nuclear medicine physician to assess the indication for the scan adequately, as well as to interpret the images.

The patient is also questioned (if possible) for relevant information that may help interpret the findings.

In pre-imaging reporting it is useful to document:

1. Serum intact PTH level and either albumin corrected or ionized calcium and serum 25-hydroxy vitamin D level (mandatory)
2. Serum phosphorus, 24-h urinary calcium levels, creatinine, markers of bone turnover, e.g., bone-specific alkaline phosphatase (optional)
3. Results of cervical US, including the thyroid and parathyroid glands
4. Results of other imaging modalities, such as 4D-CT, and MRI, if any
5. History of any prior neck and/or thoracic surgery
6. History of malignancy, because of possible tracer accumulation in neoplastic tissue
7. History of recent nuclear medicine procedures
8. Recent iodine intake (e.g., intravenous contrast medium, iodine-containing medication …) or thyroid hormone replacement therapy in case of subtraction study with Na[^99m^Tc]TcO_4_ or Na[^123^I]I, or therapy with cinacalcet and other calcimimetics in case of [^99m^Tc]Tc-MIBI imaging
9. Use of calcium channel blockers if scintigraphy is performed with [^99m^Tc]Tc-MIBI

Patients should be provided with information on how the examination is performed and the estimated duration. The patient should be advised to stay calm during image acquisition. In rare cases, e.g., in patients with severe claustrophobia, sedation may be helpful.

Patients may eat, drink, and take most of the necessary medications. However, active D vitamin analogues and calcimimetics may decrease [^99m^Tc]Tc-MIBI uptake [44]. If possible, these should be paused for 2 weeks before scintigraphy [36].

Calcium channel blockers may reduce uptake of [^99m^Tc]Tc-MIBI as well [45]. Friedman et al. in a retrospective study found that the odds ratio for a negative scan is 2.88 in patients taking calcium channel blockers compared with those without such medication (OR 2.88, 95% CI, 1.03–8.10; *p* = 0.045). Still, further studies are necessary to confirm the reversibility of this finding, and to determine the appropriate withdrawal period.

In a dual-tracer study, the thyroid scan should be performed without iodine saturation. Many factors can influence thyroid uptake of subtraction radiopharmaceuticals [46]. Dual-phase [^99m^Tc]Tc-MIBI scintigraphy, instead of dual-tracer scintigraphy, is recommended for patients on thyroid hormone replacement therapy to avoid therapy withdrawal and subsequent development of hypothyroidism.

Currently, no standardized patient preparation or image acquisition guidelines exist for parathyroid imaging with PET tracers. In the case of radiolabelled choline, vigorous exercise may increase muscle uptake of the radiopharmaceutical, so it is advisable to avoid such before scanning [47].

If a diagnostic CT scan with iodinated contrast medium is required, it is important to ask the patient about possible allergies to iodinated contrast medium as well as to ascertain an adequate kidney and thyroid function; for more information, we advise to consult the European Society of Urogenital Radiology (ESUR) guidelines on contrast media: Post-contrast acute kidney injury—Part 1: Definition, clinical features, incidence, role of contrast medium and risk factors; Post-contrast acute kidney injury—Part 2: risk stratification, role of hydration and other prophylactic measures, patients taking metformin and chronic dialysis patients [48, 49].

## Scintigraphy and SPECT

Parathyroid gland scintigraphy has involved many different radiotracers used with different protocols over the past decades.

In the 1980s, parathyroid gland scintigraphy was performed with [^201^Tl]thallous-chloride ([^201^Tl]TlCl) in combination with [^99m^Tc]Pertechnetate ([^99m^Tc]NaTcO_4_) as a dual-tracer method. The success rate in operated patients was comparably good, reaching 92% [50]. However, [^201^Tl]TlCl has poor physical characteristics as an imaging agent, due to suboptimal photon energy (69–81 keV). Additionally, it has a long physical half-life (73 h) and thus results in a nowadays unacceptably high whole-body radiation exposure. [^201^Tl]TlCl should no longer be used for parathyroid imaging. Furthermore, it is a cyclotron-produced radionuclide, and as such relatively expensive. In 1989, Coakley et al. reported the use of [^99m^Tc]Tc-MIBI for parathyroid localization, revealing better imaging characteristics of this tracer compared with [^201^Tl]TlCl *[*51*]*. Eventually, [^201^Tl]TlCl was replaced with ^99m^Tc-labeled tracers of better physical characteristics: more favorable energy (140 keV) and shorter physical half-life (6 h), resulting in lower radiation exposure. Besides, ^99m^Tc is ubiquitously available and comparatively inexpensive.

More widespread use of [^99m^Tc]Tc-MIBI and [^99m^Tc]Tc-tetrofosmin began in the 1990s. The former was used in the single-tracer and dual-tracer methods, while the latter was used mainly in a dual-tracer method in combination with Na[^99m^Tc]TcO_4_ or Na[^123^I]I.

### Radiopharmaceuticals targeting parathyroid glands

#### [^99m^Tc]Tc-hexakis-(2-methoxy-2-isobutyl isonitrile) ([^99m^Tc]Tc-MIBI)

[^99m^Tc]Tc-MIBI is the most widely used radiopharmaceutical for parathyroid scintigraphy. It is a lipophilic cationic complex that accumulates in hyperfunctioning parathyroid glands due to their increased number of mitochondria [52], which is mainly related to the number of oxyphil cells [53]. Its uptake and retention depend also on the cell cycle phase, parathyroid blood supply, capillary permeability, serum calcium level, expression of P-glycoprotein, and multidrug resistance (MDR) associated protein [54].

[^99m^Tc]Tc-MIBI has a greater uptake per gram of parathyroid tissue than per gram of thyroid tissue [55]. Additionally, it usually washes out faster from the thyroid than from the hyperfunctioning parathyroid, making it convenient for the single-tracer dual-phase method.

However, not all hyperfunctioning parathyroid glands retain [^99m^Tc]Tc-MIBI and thyroid tissue sometimes does not rapidly washout [56]. Therefore, in some cases, other protocols might be helpful. In the case of a rapid parathyroid washout, it would be reasonable to perform additional images between the standard early and late phase ones. Alternatively, dynamic imaging may be used. On the other hand, in the case of a slow thyroidal washout, subtraction imaging may be helpful.

[^99m^Tc]Tc-MIBI SPECT/CT scintigraphy is the current standard method for detecting hyperfunctioning parathyroid glands, with a reported patient-based and lesion-based pooled detection rate (DR) of 88% in a meta-analysis of twenty-three papers including 1236 patients with pHPT [57]. Furthermore, it was shown to be a cost-effective technique [58].

However, its sensitivity is significantly lower in the case of MGD compared with single-gland disease [59, 60], which is a known issue also with other imaging modalities. Nichols et al. in a study of 651 patients with HPT (20% with MGD, and 80% with single-gland disease) who underwent preoperative dual-tracer [^99m^Tc]Tc-MIBI/Na[^99m^Tc]TcO_4_ scintigraphy found the sensitivity of this method to be 61% for MGD, vs. 97% for single-gland disease [60]. Medas et al. in a study that included 212 patients with HPT (8% with MGD, 91.5% with single-gland disease, and 0.5% unknown) reported a sensitivity of [^99m^Tc]Tc-MIBI for MGD of 26.7% vs. 92.6% for single-gland disease [59].

The administered intravenous radioactivity in adults ranges from 400 to 900 MBq, depending on the patient’s body mass, whether or not SPECT is scheduled, and local regulations (Table 1). Whenever SPECT imaging is combined with CT, care should be taken to use the lowest CT dose that is compatible with the purpose.

**Table 1:** Radiopharmaceuticals for parathyroid imaging. Characteristics, recommended activity range, and effective doses

Radiopharmaceutical	Administration route	Administered activity in adults (MBq)	Photopeak energy (keV)	Physical half-life	Effective dose per activity unit (mSv/MBq)	Effective dose for the upper administered activity (mSv)
Na[^99m^Tc]TcO_4_	i.v.	74–150	140	6.04 h	0.0159*	2.4
[^99m^Tc]Tc-MIBI	i.v.	400–900	140	6.04 h	0.00703*	6.3
[^99m^Tc]Tc-tetrofosmin	i.v.	400–900	140	6.04 h	0.00629*	5.7
Na[^123^I]I	o.a. (or i.v.)	7.4–14.8	159	13.2 h	0.108* (thyroid uptake 15%)	1.6 (thyroid uptake 15%)
[^18^F]FCH	i.v.	100–300	511	110 min	0.020**	6.0
[^11^C]MET	i.v.	370–1100	511	20.3 min	0.00549*	6.0
[^11^C]CH	i.v.	200–650	511	20.3 min	0.0044***	2.9

*i.v.*, intravenous; *o.a.*,oral administration,* [163],** [164],*** [165]

#### [^99m^Tc]Tc-1,2-bis[bis (2-ethoxyethyl) phosphino] ethane) ([^99m^Tc]Tc-tetrofosmin)

[^99m^Tc]Tc-tetrofosmin is a lipophilic cation that localizes in thyroid and parathyroid tissue. Because there is no differential washout between these tissues, it is suitable only for a dual-tracer subtraction method. The mechanism of uptake is similar to [^99m^Tc]Tc-MIBI. It diffuses passively through the cell membrane and accumulates in mitochondria of parathyroid lesions [61]. The intravenously administered activity in adults ranges from 400 to 900 MBq, depending on the patient’s body mass, whether or not SPECT is scheduled, and local regulations.

However, its use in parathyroid imaging is limited, despite an overall good performance similar to that of [^99m^Tc]Tc-MIBI [56, 62, 63], owing, e.g., to the need for the dual-tracer acquisition as well as local licensing and reimbursement issues.

### Subtraction radiopharmaceuticals

#### [^99m^Tc]pertechnetate (Na[^99m^Tc]TcO_4_)

^99m^Tc has a physical half-life of 6 h and emits gamma rays of 140 keV. Na[^99m^Tc]TcO_4_ is used in the dual-tracer method as it is taken up by functioning thyroid cells. The Na[^99m^Tc]TcO_4_ image is subtracted from the [^99m^Tc]Tc-MIBI / [^99m^Tc]Tc-tetrofosmin image, thus revealing hyperfunctioning parathyroid glands.

For further details on administered activity and image acquisition, please refer also to the EANM practice guideline/SNMMI procedure standard for RAIU and thyroid scintigraphy [46]. The administered activity depends mainly on which tracer is applied first, i.e., if the thyroid study is performed before or after the [^99m^Tc]Tc-MIBI / [^99m^Tc]Tc-tetrofosmin scan. If imaging starts with Na[^99m^Tc]TcO_4_ scintigraphy, lower activities are required, i.e., 74–111 MBq compared with 150 MBq if the thyroid scan is performed after the [^99m^Tc]Tc-MIBI scan.

#### [^123^I]sodium-iodide (Na[^123^I]I)

^123^I has a half-life of 13 h and emits gamma radiation of 159 keV. Na[^123^I]I is trapped but also organified by functioning thyroid tissue. Na[^123^I]I is used in the dual-tracer method for subtraction purposes and provides good quality images. The Na[^123^I]I image is subtracted from a [^99m^Tc]Tc-MIBI / [^99m^Tc]Tc-tetrofosmin image for easier visualization of remaining hyperfunctioning parathyroid gland tissue. Disadvantages are its higher price compared with Na[^99m^Tc]TcO_4_ and the 2 h waiting time between administration of the radiopharmaceutical and acquisition of the scan. For further information on this radiopharmaceutical, we refer to the EANM practice guideline/SNMMI procedure standard for RAIU and thyroid scintigraphy [46].

### Acquisition

#### Dual-phase parathyroid scan

[^99m^Tc]Tc-MIBI scintigraphy is performed at two time points, i.e., at 10–15 min and 90–150 min after intravenous administration of the radiopharmaceutical. The administered activity ranges from approximately 400 to 900 MBq, depending on the patient’s body mass, whether or not SPECT is scheduled, and local regulations. Imaging is performed using large field-of-view gamma cameras with low-energy, high resolution (LEHR) collimators. The energy window should be in the range of 140 ± 10 keV and a matrix size of 128 × 128 or better 256 × 256 should be employed. The entire neck and the chest down to the base of the heart should be included in the field of view. It is possible to use planar images in anterior and right and left anterolateral positions, but SPECT images, especially when fused with a simultaneously acquired CT, are superior for anatomical localization of parathyroid tissue [64], especially in case of ectopic glands and altered neck anatomy [65].

SPECT/CT with [^99m^Tc]Tc-MIBI is superior to planar or stand-alone SPECT studies and dual-phase acquisition is more accurate than single-phase acquisition [66]. Therefore, it is recommended to perform at least one SPECT(/CT) study covering the area between the skull base and the heart base.

#### Dual-tracer parathyroid scan

[^99m^Tc]Tc-MIBI usually has a differential washout between the thyroid and parathyroid, so it is used as the single tracer of dual-phase parathyroid scintigraphy in many nuclear medicine departments. However, sometimes the tracer washes out rapidly from the parathyroid or is retained in the thyroid, especially in thyroid nodules. In such cases, the use of the dual-tracer method may be helpful.

##### Dual-tracer [^99m^Tc]Tc-MIBI /Na[^99m^Tc]TcO_4_ protocol

Either tracer, [^99m^Tc]Tc-MIBI or Na[^99m^Tc]TcO_4_ can be injected first. The administered activity of Na[^99m^Tc]TcO_4_ is 74–111 MBq if the protocol starts with thyroid imaging. For further details, please refer to the EANM practice guideline/SNMMI procedure standard for RAIU and thyroid scintigraphy [46]. 150 MBq of activity is used if thyroid imaging is performed after the [^99m^Tc]Tc-MIBI scan.

Na[^99m^Tc]TcO_4_ images are obtained 20–30 min after administration of the radiopharmaceutical, while [^99m^Tc]Tc-MIBI images are obtained 10–15 min after injection. Images are inspected visually, normalized, and Na[^99m^Tc]TcO_4_ images are either digitally or cognitively subtracted from the [^99m^Tc]Tc-MIBI images. The following images are obtained: - a large field of view of the neck and mediastinum (from the skull base to the heart base); − a pinhole view of the thyroid bed region; and a SPECT(/CT) acquisition whenever available.

In order to reduce administered activities and radiation exposure, a waiting time of 2–3 days between tracers may be recommended.

Imaging is performed using a large field-of-view gamma camera with LEHR collimators, a matrix size of 128 × 128 or 256 × 256, and a 15% or 20% energy window centered at the 140 keV (^99m^Tc) photon peak.

##### Dual-tracer [^99m^Tc]Tc-MIBI / Na*[*^*123*^*I]I* protocol

Na[^123^I]I is first administered orally or intravenously, and [^99m^Tc]Tc-MIBI is injected 2 h later. Na[^123^I]I and [^99m^Tc]Tc-MIBI images are acquired simultaneously, starting 5 min after injection of [^99m^Tc]Tc-MIBI. Images are inspected visually, normalized to thyroid counts, and Na[^123^I]I images are subtracted from the [^99m^Tc]Tc-MIBI images. The following images are obtained: - a large field of view of the neck and mediastinum (from the skull base to the heart base); − a pinhole view of the thyroid bed region; − a SPECT(/CT) acquisition when available [67–69].

Imaging is performed with a large field-of-view gamma camera equipped with LEHR collimators. A matrix size of 128 × 128 or 256 × 256 is recommended. For simultaneous acquisition, a 10% energy window centered on the 159 keV is recommended for Na[^123^I]I and a 15–20% window centered around 140 keV for [^99m^Tc]Tc-MIBI. Alternatively, symmetric and asymmetric windows can be used (140 keV ± 7% for [^99m^Tc]Tc-MIBI and 159 keV −4%; +10% for Na[^123^I]I) [70]. For Na[^123^I]I thyroid imaging, it is also possible to use a dedicated thyroid camera with a small field-of-view.

In both dual-tracer protocols images are subtracted at the point of similar activity of [^99m^Tc]Tc-MIBI in the thyroid gland and surrounding soft tissue.

Dual-tracer scintigraphy with subtraction imaging has an excellent performance in the detection and localization of hyperfunctioning parathyroid glands [71]. Woods et al. in a recent study reported high sensitivity and specificity for subtraction SPECT/CT (95% and 89%, respectively) [72]. The positive predictive value (PPV) and negative predictive value were 97% and 83%, respectively. The accuracy in detecting and localizing parathyroid adenomas were 94% and 92%, respectively. However, this technique adds additional radiation exposure to the thyroid; it might take additional time and is more expensive compared with a single-tracer method, although some institutions are trying to reduce administered activity and procedure length.

### Image analysis

In dual-phase scintigraphy, early and delayed images are inspected visually. Increased or sustained uptake on the delayed [^99m^Tc]Tc-MIBI image, compared with the early image, is considered a positive finding for a hyperfunctioning parathyroid gland. However, some glands show a fast washout and might not be identified clearly on delayed images. A fast clearance is more common in hyperplastic glands. SPECT images may reveal parathyroid glands that are not seen on planar images. Additionally, SPECT/CT imaging provides a superior anatomic localization of lesions.

In subtraction scanning, images need to be inspected visually before and after subtraction. Focal accumulation of radiopharmaceutical adjacent to the thyroid persisting after subtraction is suspicious for a hyperfunctioning parathyroid gland in loco *typico*; ectopic focal uptake is suspicious for an ectopic parathyroid lesion. As stated above, SPECT and SPECT/CT yield an increased sensitivity, specificity, and more precise anatomic localization compared with planar images.

The CT part of the study should be also analyzed for important findings, even without corresponding tracer uptake.

False-positive and false-negative results are occasionally encountered. The most common causes of false-positive results are inflammatory thyroiditis, cervical lymphadenopathy, and thyroid nodules (benign and malignant) behaving like parathyroid adenomas (in a single-tracer protocol) [73]. False-negative results usually occur due to small-sized hyperfunctioning glands [73], a lack of oxyphil cells [74], parathyroid hyperplasia, multiglandular disease, and high expression of P-glycoprotein [75].

## Positron emission tomography

The higher resolution of PET/CT imaging could improve the detection of the smallest pathological glands, not visualized by SPECT(/CT). It is recommended to use the scanner with the highest system sensitivity and reconstruction protocols optimized for small lesion detection (BSREM instead of OSEM). Whenever PET imaging is combined with CT, care should be taken to use the lowest CT dose that is compatible with the purpose. Several different tracers have been studied with different protocols and results.

Currently, no standardized image acquisition guidelines exist for PET tracers for parathyroid imaging.

### Radiopharmaceuticals

#### ^18^F-labeled choline analogues

Choline is a cellular proliferation marker. The mechanism of uptake of ^18^F-labeled or ^11^C-labeled choline analogues is not fully understood. As choline is a precursor of phosphatidylcholine, a phospholipid constituent of the cell membrane, its uptake, and processing for construction and maintenance of the cell membrane is a possible explanation for its accumulation in hyperfunctioning parathyroid glands [76, 77]. Stimulation of phospholipid-dependent choline-kinase results in increased radiolabeled choline uptake and is related to the secretion of PTH in HPT [78].

A commonly used choline analogue in clinical practice is (N-[(^18^F)fluoromethyl]-2-hydroxy-N,N-dimethylethanaminium ([^18^F]FCH or [^18^F]fluorocholine). Other similar radiofluorinated choline derivatives are also available and supposedly show similar clinical behavior and utility. Administered activity ranges from 100 to 300 MBq or 1.5–3.2 MBq/kg of body mass. According to the study by Rep et al., the recommended imaging time is one hour after the administration of the radiopharmaceutical, and, if possible, preceded by imaging acquired 5 min after the injection (starting as dynamic acquisition and followed by static), as some lesions may show uptake in the early phase only [79]. One acquisition 20 min after injection with delayed images in case of a negative result is also an option [40]. The field of view can be limited from the nose to the chest down to the base of the heart as for scintigraphy, to obtain a low radiation exposure from CT. But thanks to the more rapid acquisition with PET than with SPECT it may easily be extended to the whole torso or the whole body according to the patient’s history (neoplasia, skeletal disease, infection…) or incidental findings. On PET/CT machines with incremental acquisition and time of flight imaging, at least 2 min per bed position may be recommended. PET/CT with ^18^F-labeled choline analogues yields promising results, with DRs exceeding 90%.

Treglia et al. in a meta-analysis of 14 choline studies (containing 12 studies with ^18^F-labeled and 2 with ^11^C-labeled choline derivatives) involving 517 patients, reported pooled values for the sensitivity of 95%, PPV 97%, and DR 91% on a per-patient analysis. In a per-lesion analysis, the pooled sensitivity was 92% and PPV was 92% [78].

In addition, Hocevar et al. in a recent study demonstrated that patients with a single adenoma on [^18^F]fluorocholine PET/CT can safely and accurately undergo minimally invasive parathyroidectomy without intraoperative PTH testing [80]. Preoperative localization was reliable in 96.8% of patients.

In a recent head to head comparative study including 103 patients with pHPT, the diagnostic performance of [^18^F]fluorocholine PET/CT was superior to conventional scintigraphic methods ([^99m^Tc]Tc-MIBI SPECT/CT, [^99m^Tc]Tc-MIBI / Na[^99m^Tc]TcO_4_ subtraction imaging, and [^99m^Tc]Tc-MIBI dual-phase imaging), separately or combined, with a sensitivity of 92% for [^18^F]fluorocholine PET/CT, compared with 39–56% for conventional imaging, and 65% for a combination of conventional methods [81]. The impact of the performance of the PET/CT machine on the performance of [^18^F]fluorocholine imaging in patients with negative or inconclusive [^99m^Tc]Tc-MIBI SPECT/CT has been recently assessed by Lopez-Mora et al. [82]. In a prospective series of 33 patients with pHPT confirmed at surgery, they found that [^18^F]fluorocholine PET/CT employing an analogic scanner could detect hyperfunctioning tissue in 22 of 33 of patients with negative or inconclusive [^99m^Tc]Tc-MIBI SPECT/CT, while a digital scanner detected hyperfunctioning tissue in 30 of 33 patients. The lesions detected only by the digital system were < 10 mm in diameter on resection, highlighting the potential importance of new PET/CT technologies in the detection of small parathyroid lesions.

Other advantages of PET/CT with ^18^F-labeled choline analogues over [^99m^Tc]Tc-MIBI scintigraphy are the lower radiation exposure [83], the higher resolution, and the shorter acquisition time. It is therefore considered an alternative first-line imaging method [84, 85].

Potential disadvantages of PET/CT with ^18^F-labeled choline analogues are higher costs, the uptake by inflammatory lymph nodes and thyroid nodules as a potential source of false-positive results [86], and local reimbursement and licensing issues.

^18^F-labeled choline analogues may be also used in PET/MRI systems. This novel method is promising in patients with inconclusive results of standard imaging techniques [87].

#### [^11^C]2-hydroxy-N,N,N-trimethylethanamium ([^11^C]CH)

As mentioned above, choline is a precursor of phosphatidylcholine, which may explain the fact that [^11^C]CH shows strong uptake in hyperfunctioning parathyroid cells [76].

Noltes et al. tried to optimize the protocol for [^11^C]CH PET/CT imaging, taking into consideration the activity and image quality [88]. They retrospectively studied 21 patients with pHPT and suggested image acquisition 20 min after administration of 6.3 MBq/kg [^11^C]CH, with a scanning time of at least 5 min. Orevi et al. in a prospective pilot study including 40 patients with pHPT indicated that [^11^C]CH provides higher image quality than [^99m^Tc]Tc-MIBI, with the same or even superior diagnostic accuracy [89].

The advantage of [^11^C]CH PET/CT over scintigraphy is a shorter acquisition time. There is fast uptake of [^11^C]CH in hyperfunctioning parathyroid tissue, as well as a rapid clearance of background activity. Imaging is completed within 30 min. In addition, the radiation exposure is lower. The short physical half-life of ^11^C (20.3 min) results in significantly lower radiation exposure than using a ^18^F-labeled choline analogue, as well as other nuclear medicine techniques and 4D-CT.

On the other hand, the short physical half-life is a drawback, restricting the use of ^11^C to facilities with an on-site cyclotron. Additionally, a higher average positron energy of ^11^C leads to more noise on the images and poorer spatial resolution compared with ^18^F.

#### l-[methyl-^11^C]methionine ([^11^C]MET)

[^11^C]MET is a PET radiopharmaceutical that was previously often used as a second-line imaging tracer after negative or inconclusive conventional imaging. It is trapped in the hyperfunctioning parathyroid gland as it is involved in the synthesis of the PTH precursor [90]. The administered activity of the radiopharmaceutical varies between 370 and 1100 MBq in different [^11^C]MET studies. Reported acquisition time is also highly variable, ranging from a start immediately after to 40 min after injection. In most studies, PET scanning is preceded or followed by a low-dose CT scan. Some authors recommend imaging at two time points, 10 min and 40 min after application of the radiopharmaceutical, preceded or followed by CT scanning for attenuation correction [90, 91]. The best parathyroid to cervical soft-tissue ratio was accomplished 10 min and the best parathyroid to thyroid ratio 40 min after administration of the radiopharmaceutical [90].

Increased uptake at early or late imaging is considered positive.

Kluijfhout et al. in a meta-analysis of 14 [^11^C]MET studies found a pooled sensitivity of 77% and a pooled PPV of 98% for the detection of hyperfunctioning parathyroid glands in the correct quadrant [92]. There was no difference in sensitivity in patients with and without previous negative or inconclusive standard imaging (81% vs. 78%, respectively). However, Noltes et al. in a recent retrospective study found that [^11^C]MET PET/CT is able to identify parathyroid lesions on the correct side in only 64% (18/28) of patients after prior negative [^99m^Tc]Tc-MIBI SPECT/CT and/or cUS [93].

Weber et al. prospectively studied whether [^11^C]MET PET/CT is able to identify [^99m^Tc]Tc-MIBI-negative hyperfunctioning parathyroid glands in a cohort of 50 pHPT patients [94]. Pre-surgical scanning with [^11^C]MET PET/CT localized hyperfunctioning parathyroid glands in 74% of patients with negative [^99m^Tc]Tc-MIBI scintigraphy. [^11^C]MET PET/CT detected 40 out of 57 (70%) hyperfunctioning glands.

The major limitations of this radiopharmaceutical for more widespread use are the short physical half-life of ^11^C (20.3 min) which necessitates an on-site cyclotron, the demanding labeling process which requires an on-site radiopharmacy with competent radiochemical staff, and a higher average positron energy which leads to more noise compared with ^18^F. Additionally, there are local reimbursement and licensing issues.

#### Other PET tracers

Other PET tracers are not recommended for the detection of hyperfunctioning parathyroid glands, based on currently published data. ^18^F-fluoro-D-glucose ([^18^F]FDG) is almost ubiquitously available and has the advantage of possible off-site usage due to a significantly longer physical half-life of ^18^F (110 min) compared with ^11^C. However, sensitivity and PPV vary considerably between the few available studies, 0–94% and 62–100%, respectively. Therefore, this tracer does not seem suitable for the detection of hyperfunctioning parathyroid glands compared with other standard imaging techniques [92]. However, sometimes incidental parathyroid adenomas may be detected in [^18^F]FDG studies acquired for other reasons than HPT. 3,4-dihydroxy-6-[^18^F]fluoro-L-phenylalanine (6-[^18^F]F-DOPA) does not appear promising, as reported by Lange-Nolde et al. [95]. These authors scanned 8 patients with pHPT and histologically proven parathyroid adenomas, but none of the patients showed any detectable uptake of 6-[^18^F]FDOPA in adenoma. O-(2-[^18^F]fluoroethyl)-L-tyrosine ([^18^F]FET) also does not appear to be promising as reported by Krakauer et al. [96]. In two patients, dual-isotope SPECT/CT located a parathyroid adenoma, verified by histopathology; only faint uptake of [^18^F]FET was detected in the hyperfunctioning parathyroid glands. The maximum (but low) target-to-background ratio was reached 30 min after tracer administration.

### Image analysis

The reading of PET/CT or PET/MRI to localize hyperfunctioning parathyroid gland(s) usually starts with visualizing multiple intensity projections which may show foci behind the thyroid gland and help in detecting ectopic foci. The analysis of attenuation-corrected slices in the 3 planes with and without CT (or MRI) fusion is necessary in all cases, to precise the anatomical landmarking and the size of abnormal foci. The analysis of non-attenuation-corrected images may sometimes be useful in the case of artifacts on CT or of incidental foci in the lungs. For the neck region, the soft-tissue CT window should be used. A reading of PET and PET/CT (MRI) fused images of organs or structures out of the neck but included in the whole field of view, should not be omitted. The soft-tissue CT window is adapted to the mediastinum and the breasts (and the liver in the absence of a specific window), but other CT windows are recommended for lung and bone (and liver if this window is available).

All foci located from the upper neck to the base of the heart with an increased tracer uptake compared with the adjacent background should be considered. Focal accumulation of radiopharmaceutical in a nodule next to the thyroid is suspicious for a hyperfunctioning parathyroid gland in the *loco typico*. A hypodense character compared with the thyroid parenchyma on CT and a SUVmax greater than that of the thyroid parenchyma are further arguments favoring this interpretation [35]. In the case of dual-time point acquisition, the persistence in the late images of foci visible on the early images is a further argument in the case of equivocal early findings, but the disappearance of an evocative focus on early images could be due to a hyperfunctioning parathyroid gland with rapid clearance of the tracer. Even though the thyroid uptake of choline-based tracer is usually moderate, it is diffusely intense in case of thyroiditis or of Graves’ disease, making the detection of foci adjacent to the thyroid gland difficult. The main interpretation problem is a focal tracer uptake inside the thyroid which corresponds in most cases to a malignant or benign thyroid nodule (in particular oxyphil adenomas are [^18^F]FCH avid) but may also correspond to an intrathyroidal parathyroid gland [35]. In methionine studies, thyroid nodules may also cause false-positive results [93]. In case of thyroid anomalies, it is recommended to analyze the PET/CT images with reference to thyroid scintigraphy (with Na[^123^I]I if available) to check how PET/CT foci match with the location and the iodine-metabolism of thyroid nodules; maximum intensity projection and coronal slices are particularly useful in this aim. Small thyroid remnants in case of partial or total thyroidectomy may take up the PET tracer leading to a false-positive result which may be avoided by referring to thyroid scintigraphy. Concerning the interpretation of ectopic foci, the main pitfall with metabolic tracers is uptake by reactive lymph nodes. In particular [^18^F]FCH is very frequently taken up by mediastinal and/or axillary lymph nodes which are not suspicious for ectopic parathyroid glands. Uptake by one single cervical lymphadenopathy may constitute a problem for interpretation [86]. The analysis of the CT (or MRI) component yields a more precise anatomical localisation and may also increase specificity.

False-negative results in choline/methionine studies may occur in cases of moderate hypercalcemia and non-elevated PTH serum levels (so-called “abnormally normal serum PTH level). In the case of MGD, one or several hyperplastic glands may be missed, particularly in sHPT or MEN. False-negative results may also be caused by a too restricted field of view, in particular in case of recurrence after parathyroidectomy.

## Reporting parathyroid radionuclide imaging

The scan report should include patients’ relevant identifying data as well as the relevant clinical history including serum PTH and calcium levels and any further relevant details, such as a history of MEN or history of (para)thyroid surgery.

The report should include a technical section, mentioning the protocol employed, the tracer(s) used together with administered activities and route of administration, which images were acquired (planar projections, SPECT/CT, PET/CT, PET/MRI, including the anatomic area), the dose-length product if CT has been acquired, and their respective timing and, if relevant, details on image reconstruction.

Reports need to include at least the description of abnormal findings (one or more hyperfunctioning parathyroid lesions) and localization of abnormal gland(s) expressed in terms of the four thyroidal quadrants including the gland(s) depth, or the particular ectopic location, as well as the images in which these are identified. Detection of a hyperfunctioning parathyroid gland with [^99m^Tc]Tc-MIBI, whose implantation in the forearm had not been recorded, was reported [97].

Additionally, one should document any incidental finding, such as pathological [^99m^Tc]Tc-MIBI uptake in the pituitary region (if included in the field of view) [98], or in the lungs [99], or ^18^F-labeled choline analogues foci suspicious for malignancy [100, 101], which should prompt further diagnostic investigation. A frequently abnormal bone uptake of [^99m^Tc]Tc-MIBI has been reported by Zhao et al. in 22 (27.8%) patients [102]. Nineteen of them showed diffusely increased activity in the skeleton, 2 patients had focal uptake in brown tumors, and one showed both of these patterns. Brown tumors, also known as osteitis fibrosa cystica, are a specific complication of HPT, mostly in the case of sHPT or prolonged pHPT, or parathyroid carcinoma. They are usually multiple, but also a solitary lesion may be present [103]. On CT, brown tumors appear as lytic or multilobular cystic changes. They may be detected with ^18^F-labeled choline analogues whereas [^99m^Tc]Tc-MIBI is negative [104]. In the case of incidental bone foci, this etiology may be considered as well as primary or secondary bone malignancies [105–107]. Diffuse and intense tracer uptake in the jaw may be observed in rare cases, being evocative of HPT-jaw tumor syndrome, a genetic disease linked with a negative prognosis [108–110].

## Cervical ultrasonography

It is recommended to perform cUS in conjunction with nuclear medicine imaging. Cervical ultrasonography is often used for parathyroid gland localization. It is also utilized for confirming the diagnosis of the hyperfunctioning parathyroid gland(s) by guided FNA cytology and analysis of the FNA-PTH in highly selected cases. It also provides an additional evaluation of the thyroid that may change surgical management, especially in the case of coexisting (suspected) malignant nodules [111].

Cervical ultrasonography is a low-cost, non-invasive (apart from guided FNA), accurate, and highly sensitive technique in experienced hands [112].

It is performed with the patient in a supine position with a hyperextended neck, using a high-frequency (≥ 7 MHz) linear transducer. It is recommended to perform cross-sectional and longitudinal images of the anterior neck region, between the common carotid arteries, from the level of the carotid bifurcation down to the superior mediastinum, particularly paying attention to the posterior surface of the thyroid and the region below it. The size and location of the thyroid, thyroidal lesions, and any suspected parathyroid glands should be carefully documented. Owing to their small size (approximately 3 to 5 mm), normal parathyroid glands are usually not identified on cUS. Hyperfunctioning parathyroid glands are detectable due to their size and echogenicity. They are usually hypoechoic, circumscribed, ovally shaped, delineated from the thyroid by hyperechoic connective tissue. However, they may have various shapes and contain fluid and, rarely, calcifications. Some glands have undergone cystic degeneration, presenting as anechoic lesions with dorsal echo amplification. Sometimes, a peripheral artery branching into the gland can be seen.

Occasionally it may be difficult to differentiate enlarged parathyroid glands from lymph nodes [33].

Cheung et al. in a meta-analysis of 19 studies found that cUS has a pooled sensitivity of 76.1% and a PPV of 93.2% in the preoperative localization of enlarged parathyroid glands in pHPT patients [113]. The diagnostic accuracy of ultrasound may be improved when performed after radionuclide imaging [114].

Cervical ultrasonography also has some limitations. It cannot detect ectopic parathyroid glands below the neck level VI and is less sensitive in the detection of small glands or MGD. Ruda et al. in a systematic review of 20,225 cases of pHPT found that the sensitivity of cUS in patients with two adenomas was 16.2% and in MGD 34.9%, compared with 78.5% in single-gland disease [115]. Medas et al., in a retrospective study of 212 patients, also reported lower cUS sensitivity in patients with MGD compared with patients with single-gland disease (18.7% and 66.5%, respectively) [59]. Haciyanli et al. reported accuracy of cUS of 40% in patients with two adenomas, while the combination of cUS and [^99m^Tc]Tc-MIBI yielded an accuracy of 60% [116].

False-negative results also occur with an intrathyroidal location of hyperfunctioning parathyroid glands, being misinterpreted as thyroid nodules. Fine-needle aspiration cytology accompanied by the measurement of the FNA-PTH may solve this diagnostic challenge [117]. Furthermore, enlarged parathyroid glands adjacent to the upper thyroid poles may have a similar echogenicity as the thyroid gland and might hence be missed [112].

Medas et al. reported a lower sensitivity in patients with concomitant thyroid disease compared with patients with normal thyroid tissue (46.7% vs. 75%) [59]. Thyroid nodules are a common reason for false-positive results, especially if located posteriorly [118]. This is an important issue since synchronous thyroid pathology is common in pHPT patients (18% as reported by Bentrem et al.) [119].

For the reasons stated above, and since it is an operator-dependent technique, it is recommended to combine cUS with parathyroid scintigraphy [73].

For FNA-PTH, a sensitivity of 70–100%, and a specificity of 75–100% has been published, rendering it more reliable than cytology alone [120]. However, Trimboli et al. have shown that despite satisfactory results reported, this procedure is not widely used because it is not well standardized from pre- to post-analytical aspects. There is no consensus with regard to the FNA-PTH reference range and upper cut-off value. Some authors consider any FNA-PTH positive that is above the serum PTH level [121–124], whereas others suggest an FNA-PTH/serum PTH ratio of ≥ 2:1 in order to minimize the influence of sample contamination by blood [120].

However, in the case of non-diagnostic or indeterminate cytology findings, FNA-PTH might be useful.

Fine-needle aspiration may harbor complications, such as post-FNA fibrosis of the parathyroid gland and surrounding structures, making surgery more difficult and time-consuming [125]. It may also cause bedeviled histopathology by imitating malignancy. Norman et al. conducted a study that included 30 patients who underwent FNA of a parathyroid adenoma and a control group of 3000 patients who underwent surgery without previous FNA. On histopathology, adenomas in the control group showed a fibrotic reaction in 4.3% of cases, while glands with previous FNA had a fibrotic reaction in 77% of cases. The fibrotic reaction also imitated malignancy on histopathology. Additionally, the fibrotic reaction was more common with repeated FNA passes. Fine-needle aspiration was associated with a doubling of the surgery time [125]. Rarely, FNA may cause an inflammatory reaction, parathyroid abscess, or hematoma [126]. Very rare complications of FNA include parathyromatosis [127, 128] and the potential risk of parathyroid carcinoma seeding [129, 130]. For the reasons stated above, FNA cytology is recommended only in highly selected cases, e.g., atypical cUS findings, or inconclusive scintigraphy.

## Computed tomography

Standard X-ray CT has limited value in the detection of enlarged parathyroid glands in pHPT [112].

Four-dimensional CT (4D-CT) consists of standard CT imaging with 3 vascular phases (non-enhanced, arterial, and venous), and the fourth dimension that allows enhancement evaluation over time [131, 132]. Hence, this technique also provides functional data on enlarged parathyroid glands. Fast uptake and washout of contrast are considered typical for parathyroid adenomas [73].

This technique has similar diagnostic performance compared with [^99m^Tc]Tc-MIBI SPECT [112], although protocols vary among institutions, which results in minor differences in DRs [133–135].

4D-CT has the advantage of short imaging time and is promising for the detection of small and ectopic parathyroid glands, but its drawbacks are comparably high radiation exposure and the need for iodinated contrast medium. The dose of radiation delivered to the thyroid is considerably higher than with standard imaging methods. Mahajan et al. reported a 57 times higher (92.0 vs. 1.6 mGy) thyroid dose compared with [^99m^Tc]Tc-MIBI SPECT imaging [136]. As this at least in theory results in an increased risk for thyroid malignancies, this method should be used with caution, especially in younger patients. Some institutions perform fewer acquisitions to reduce radiation exposure. However, this in turn leads to a reduction in sensitivity. Kluijfhout et al. in a meta-analysis of 34 studies (2563 patients) found a lower pooled sensitivity of a single-contrast phase protocol (71%), compared with 2- and 3-contrast phase protocols (76% and 80%, respectively) [137]. They suggested that a 2-contrast phase protocol is sufficient with regard to sensitivity and radiation exposure.

4D-CT is useful in the case of negative previous imaging studies or in patients with distorted neck anatomy. It is a good method for the preoperative detection of hyperfunctioning parathyroid glands after the unsuccessful initial surgery. Mortenson et al. in a prospective study of 45 patients who underwent parathyroid reoperation, revealed a higher sensitivity of 4D-CT compared with [^99m^Tc]Tc-MIBI SPECT/CT and neck US (88%, 54%, and 21% respectively) [138].

Combined [^18^F]fluorocholine PET and 4D contrast-enhanced CT (4DCeCT) may be considered in pHPT patients with negative or inconclusive first-line imaging, as stated by Piccardo et al. [135]. They prospectively enrolled 44 pHPT patients. [^18^F]fluorocholine PET/4DCeCT was positive in 32 of 44 patients with pHPT (72.7%), and in 31 of 31 operated patients (100%), being superior to [^18^F]fluorocholine PET/CT alone (80% and 56.8%, respectively) and 4DCeCT alone (74% and 54.5%, respectively).

However, the reported sensitivity of CT for a MGD is still as low as 32–53% [139].

## Magnetic resonance imaging

Hyperfunctioning parathyroid glands may have variable MRI characteristics. However, they usually show intermediate to low signal intensity on T1-weighted images and high signal intensity on T2-weighted images [140].

Lopez Hänninen et al. in 2000 reported a high sensitivity of MRI for identifying hyperfunctioning parathyroid glands [141]. 1.5-T MRI detected 32 out of 39 surgically proven abnormal parathyroid glands (82%). Sensitivity was higher for adenomas compared with hyperplasia (87% and 75%, respectively).

Wakamatsu et al. in a study from 2003, involving 35 patients with pHPT and 4 with sHPT, reported an unsatisfactory overall MRI sensitivity for the detection of hyperfunctioning parathyroid glands (43.4%) [142]. There were 29 patients with the single-gland disease and 10 with MGD. The sensitivity for the first group was slightly higher (48.3% vs. 37.5%, respectively). However, they used a 0.5 T MRI, which may influence sensitivity.

A recent study by Argirò et al. demonstrated the correct localization of hyperfunctioning parathyroid glands in 45/46 patients that underwent 3 T MRI with standard clinical pulse sequences, yielded an excellent preoperative diagnostic performance, with a sensitivity of 97.8% and a specificity of 97.5% [143]. Furthermore, it showed a good performance in MGD (8/8 enlarged glands detected) and ectopic parathyroid lesions (6/7 glands detected).

Merchavy et al. in a study from 2016 reported that 4D MRI is a useful technique for the preoperative detection of parathyroid lesions [144]. In 10 out of 11 patients with parathyroid lesions, 4D MRI localized adenomas correctly. The reported sensitivity was 90%, and 100% after optimizing the imaging time. The specificity was 100%.

Nowadays, MRI is mostly used as a second-line imaging technique.

While the use of gadolinium-based contrast agents is typically necessary, MRI does not carry any radiation exposure, which is a significant advantage of this technique. MRI is also used as part of PET/MRI exams with ^18^F-labeled choline analogues and is considered particularly useful for the detection of cystic adenomas with comparably low tracer uptake [145, 146].

The use of MRI is limited in patients with kidney failure (if contrast medium is used) and implanted medical devices, such as cardiac pacemakers. Both kidney failure and cardiac arrhythmia are associated with HPT [76]. Furthermore, it may also be difficult for MRI to differentiate hyperfunctioning parathyroid glands from lymph nodes [142].

Further larger studies are necessary to confirm the diagnostic performance of MRI in this setting.

## Invasive diagnostic procedures

Invasive diagnostic procedures are used in case of prior unsuccessful parathyroid surgery and negative or equivocal non-invasive imaging studies. They are rarely utilized nowadays due to improved standard non-invasive imaging and because they carry certain risks for the patient. Invasive techniques are associated with complications such as hematoma, contrast-induced nephropathy, and cerebrovascular infarction [73]. Anaphylaxis due to an allergic reaction to an iodinated contrast medium is an additional complication, also observed in non-invasive imaging (CT). Thus, invasive techniques should be performed by a highly experienced operator.

### Selective venous sampling

Selective venous sampling is the most commonly utilized technique. Serial blood samples are obtained from the superior vena cava, bilateral brachiocephalic, internal jugular, vertebral, thymic, superior, middle, and inferior thyroid veins [73]. An increase in PTH level from the vein drainage sites is compared with a peripheral sample. Ibraheem et al. in a recent meta-analysis of 12 studies found a pooled sensitivity and specificity of 74% and 41%, respectively [147]. This method had a higher pooled sensitivity compared with non-invasive methods, mainly US and SPECT tracers, in re-operated patients.

### Selective arteriography

Selective arteriography can be useful in patients with altered venous drainage due to prior neck surgery [73]. It is performed by selective transarterial induction of hypocalcemia in combination with nonselective venous sampling. An increase in PTH levels after stimulation is compared with baseline. A blush on the arteriogram is also considered a positive finding. Powell et al. in a study from 2009 found that arteriography is the best invasive procedure for localizing hyperfunctioning parathyroid glands (92% PPV), before revision surgery [148].

## Open issues

Primary hyperparathyroidism is usually caused by a solitary parathyroid adenoma, which is relatively easy to detect. However, in 15–20% of cases, it is caused by MGD, i.e., multiple adenomas or hyperplastic glands, which are more difficult to diagnose with SPECT/CT and PET/CT techniques, cUS, 4D-CT, and MRI. Additionally, in the last decades, there is a trend toward minimally invasive parathyroidectomy whenever possible, which may be another reason for missed MGD. However, in suspected MGD, not identified by standard techniques, open neck exploration should remain as an option. Another reason for failed imaging and unsuccessful surgery is an ectopic parathyroid gland. The reasons for imaging failure in this setting may be an inadequate imaging technique, failure to detect a rare ectopic site, as well as the imprecisely reported location of the ectopic gland. An additional problem, not only related to MGD and ectopic sites, is that not all hyperfunctioning parathyroid glands retain radiopharmaceuticals and the thyroid in certain cases does not show adequate washout. In these circumstances, dynamic or subtraction imaging might be helpful.

Another potential problem is a failure to detect ectopic glands in the pericardial region, due to the normal distribution of cardiotropic SPECT radiopharmaceuticals in neighboring myocardium. While these cases are extremely rare, they still need to be recognized.

Additional problems are caused by discordant imaging results. Novel techniques, such as PET/MRI with ^18^F-labeled choline analogues, appear to be promising in patients with inconclusive results of standard imaging techniques [87]. PET/MRI might be even more advantageous than PET/CT, owing to less radiation exposure and better soft-tissue contrast in the neck, improving the morphological correlation of PET findings. Current data exists from a few studies with up to 42 patients [87, 145, 146, 149], the majority of patients having negative or equivocal standard imaging.

Another rare but important issue is the imaging of parathyroid carcinoma and distinguishing it from benign parathyroid lesions. Parathyroid carcinoma is typically characterized by significant hypercalcemia and high PTH levels, but there are no specific biochemical features that allow for differentiation from benign parathyroid lesions. Several US features may be helpful in parathyroid carcinoma cases, such as larger lesion size (> 3 cm), irregular lesion borders with tissue invasion, heterogeneous and decreased echogenicity, or tumor depth/with ratio of ≥1:1 [150]. Parathyroid carcinoma, its recurrences, and minimally invasive parathyroid carcinoma, usually accumulate [^99m^Tc]Tc-MIBI [151–156], and recently it was demonstrated that parathyroid carcinoma has a higher retention level (mean and peak retention index) of [^99m^Tc]Tc-MIBI compared with benign lesions [157], which may be helpful in the preoperative differential diagnosis. PET/CT with ^18^F-labeled choline analogues is able to detect minimally invasive parathyroid carcinoma, according to recently published papers [154]. It may also detect local and distant recurrences of parathyroid carcinoma by their high uptake [158, 159]. [^18^F]FDG PET/CT may also provide information on loco-regional and distant spreading, presumably in less well-differentiated lesions [152, 160]. One isolated case of a [^99m^Tc]Tc-MIBI-negative and [^18^F]FDG-negative, but [^11^C]MET-positive parathyroid carcinoma local recurrence has been described [161]. Computed tomography and MRI are useful for detecting the local extent of disease, invasion of surrounding tissue, and distant spread. Fine-needle aspiration is not recommended in suspected parathyroid carcinoma, because cytology is not accurate in distinguishing malignant from benign lesions, and it may cause seeding of cancer cells [162].

Another issue is discussed in a recent article regarding the lack of specific criteria for the diagnosis of minimally invasive parathyroid carcinoma [154]. It appears to be less aggressive compared with “classic” parathyroid carcinoma and authors suggest the importance of differentiating it from parathyroid adenoma and parathyroid carcinoma.

## Conclusions

The use of optimized first-line imaging for the detection and precise localization of hyperfunctioning parathyroid glands is important as the first operation is the best moment to obtain a long-lasting cure. Combining [^99m^Tc]Tc-MIBI SPECT/CT with cUS performed by an experienced sonographer is a widely available and accepted first-line strategy. Different protocols exist for [^99m^Tc]Tc-MIBI scintigraphy. Dual-phase imaging with SPECT/CT is a widely used technique. Dual-tracer subtraction imaging is a valuable alternative option. PET/CT with ^18^F-labeled choline analogues has shown superior results; however, data from large cohorts and on cost-effectiveness are not currently available. It may be considered a potential “alternative” first-line method whenever possible, as it appears to be an effective technique even in patients with negative/equivocal standard imaging findings. Other advantages of this technique are the high resolution, the low radiation exposure, and the shorter acquisition time. Modern sensitive PET/CT scanners and the use of PET/MRI for ^18^F-labeled choline analogue imaging may result in significantly less radiation exposure compared with [^99m^Tc]Tc-MIBI SPECT/CT. The drawbacks are higher costs compared with [^99m^Tc]Tc-MIBI, as well as local reimbursement and licensing issues. There are several second-line techniques available after negative or inconclusive first-line imaging. It is important to obtain concordant imaging by two techniques whenever possible. [^11^C]MET is a radiopharmaceutical that may be used as a second-line PET imaging tracer as it has an excellent detection performance. However, major drawbacks of this radiopharmaceutical are the short physical half-life of ^11^C which demands an on-site cyclotron, the demanding labeling process, poorer spatial resolution, local reimbursement, and licensing issues.

4D-CT may be useful in case of negative or inconclusive other imaging studies, in patients with distorted neck anatomy, or after futile surgery. It has similar diagnostic accuracy compared with [^99m^Tc]Tc-MIBI SPECT, but a higher radiation exposure. ^18^F-labeled choline analogue PET may be combined with 4D-CT in complicated cases (e.g., re-operated patients) to enhance the sensitivity and PPV compared with either technique alone. MRI may be also used after negative/inconclusive first-line imaging or in pregnant patients. Invasive diagnostic procedures remain as last resort.

## Liability statement

This guideline summarizes the views of the EANM Thyroid Committee. It reflects recommendations for which the EANM cannot be held responsible. The recommendations should be taken into context of good practice of nuclear medicine and do not substitute for national and international legal or regulatory provisions.
